# RNAi Screen Reveals an Abl Kinase-Dependent Host Cell Pathway Involved in *Pseudomonas aeruginosa* Internalization

**DOI:** 10.1371/journal.ppat.1000031

**Published:** 2008-03-21

**Authors:** Julia F. Pielage, Kimberly R. Powell, Daniel Kalman, Joanne N. Engel

**Affiliations:** 1 Program in Microbial Pathogenesis and Host Defense, University of California San Francisco, San Francisco, California, United States of America; 2 Department of Medicine, University of California San Francisco, San Francisco, California, United States of America; 3 Department of Microbiology & Immunology, University of California San Francisco, San Francisco, California, United States of America; 4 Department of Pathology and Laboratory Medicine, Emory University, Atlanta, Georgia, United States of America; Tufts University School of Medicine, United States of America

## Abstract

Internalization of the pathogenic bacterium *Pseudomonas aeruginosa* by non-phagocytic cells is promoted by rearrangements of the actin cytoskeleton, but the host pathways usurped by this bacterium are not clearly understood. We used RNAi-mediated gene inactivation of ∼80 genes known to regulate the actin cytoskeleton in Drosophila S2 cells to identify host molecules essential for entry of *P. aeruginosa*. This work revealed Abl tyrosine kinase, the adaptor protein Crk, the small GTPases Rac1 and Cdc42, and p21-activated kinase as components of a host signaling pathway that leads to internalization of *P. aeruginosa*. Using a variety of complementary approaches, we validated the role of this pathway in mammalian cells. Remarkably, ExoS and ExoT, type III secreted toxins of *P. aeruginosa*, target this pathway by interfering with GTPase function and, in the case of ExoT, by abrogating *P. aeruginosa*–induced Abl-dependent Crk phosphorylation. Altogether, this work reveals that *P. aeruginosa* utilizes the Abl pathway for entering host cells and reveals unexpected complexity by which the *P. aeruginosa* type III secretion system modulates this internalization pathway. Our results furthermore demonstrate the applicability of using RNAi screens to identify host signaling cascades usurped by microbial pathogens that may be potential targets for novel therapies directed against treatment of antibiotic-resistant infections.

## Author Summary

Mortality from *Pseudomonas aeruginosa* infections, one of the leading causes of hospital acquired infections, approaches 40%, and multiple drug resistant infections are common and increasing. Internalization of *P. aeruginosa* by the host cell appears to play a fundamental role in the pathogenesis of this opportunistic bacterium, but the host cell factors involved in this process are incompletely understood. We used a targeted RNAi screen in Drosophila S2 cells to identify a subset of regulators of the host actin cytoskeleton that contribute to bacterial entry and confirmed their involvement in infection of mammalian cells. We found that *P. aeruginosa* can modulate this internalization pathway in a complex manner by injecting the bacterial toxins ExoS and ExoT into the host cell via its type III secretion system. The identified host cell molecules may serve as targets for novel drugs to treat infections resistant to conventional antibiotics and may be applicable to a wide range of pathogens.

## Introduction

*Pseudomonas aeruginosa* is one of the leading causes of nosocomial infections in humans. In the setting of pre-existing epithelial tissue damage and/or host immunocompromise, *P. aeruginosa* is able to cause severe infections of the respiratory and urinary tract, skin, and eye [1]. In addition, *P. aeruginosa* has a unique ability to cause chronic infections in the lungs of patients with cystic fibrosis, leading to end stage lung disease and death [2].

Like many gram-negative pathogens, *P. aeruginosa* possesses a type III secretion system (T3SS) that is critical to virulence *in vitro* and *in vivo* [1]. Through this apparatus *P. aeruginosa* secretes and translocates into the host cell bacterial effectors that subvert host cell functions. Four T3SS effectors have been identified in *P. aeruginosa*: ExoU is a potent phospholipase that causes rapid host cell death [3],[4]; ExoY is an adenylate cyclase that induces cell rounding [5]; ExoS and ExoT are highly homologous bifunctional proteins, with N-terminal GTPase activating protein (GAP) domains and C-terminal ADP ribosyltransferase (ADPRT) domains. For both ExoS and ExoT, the GAP domain targets Rho family GTPases, including Rho, Rac1, and Cdc42 [6]–[9]. In contrast, the substrate specificity of the ADPRT domains is distinct and non-overlapping [10]. While the ExoS ADPRT domain ADP ribosylates diverse proteins, such as Ras, Ral, Rabs, Rac1, and Ezrin [11]–[14], the ADPRT domain of ExoT primarily targets the SH2 domain of Crk family proteins [15],[16]. Together, the activities of these T3SS effectors are critical for initial colonization and subsequent acute damage to the mucosal barrier, in part by causing disruption of the host cell cytoskeleton, breakdown of cell-cell junctions, and inhibition of wound healing [17]–[20].

The presence and/or production of T3SS effectors are variable amongst *P. aeruginosa* strains and may account for some of the phenotypic differences observed in different isolates. Indeed, almost no strain encodes all four effectors [21]. Approximately 25% of *P. aeruginosa* strains examined thus far encode only ExoU and ExoT [21]. These strains are cytotoxic and poorly internalized by epithelial cells, however isogenic mutants lacking these two effectors more efficiently enter host cells [22]. The remaining 75% of *P. aeruginosa* strains produce only ExoS and ExoT, actin-disrupting toxins that have been shown to cause cell death and inhibit bacterial internalization [18],[23]. Despite the presence of ExoS and ExoT, these strains are efficiently internalized into epithelial cells [24]. Taken together, these observations demonstrate that all strains of *P. aeruginosa* are capable of entering host cells, suggesting a fundamental role of invasion in the pathogenesis of *P. aeruginosa* infections.

The molecular mechanisms underlying *P. aeruginosa* invasion into non-phagocytic cells, such as those that line the mucosal barrier, are incompletely understood. *P. aeruginosa* entry is an actin-dependent process that involves Rho family GTPases [25]. Recent studies suggest that Phosphatidylinositol 3-kinase (PI3K) and its effector Protein kinase B/Akt, which act both upstream and downstream of Ras and Rho family GTPases [26], are necessary for and activated upon internalization of *P. aeruginosa* into Madin Darby Canine Kidney (MDCK) cells [27]. *P. aeruginosa* entry also leads to activation of tyrosine kinases, such as Src [28],[29], and subsequent tyrosine phosphorylation of several host proteins, including Caveolin [30]. Some strains of *P. aeruginosa* are internalized through activation of acid sphingomyelinase and the release of ceramides in sphingolipid-rich rafts [31]. Reorganization of these rafts into larger signaling platforms is required for internalization of bacteria, induction of apoptosis, and the regulation of the cytokine response in infected cells [31].

While these studies are informative, a comprehensive understanding of *P. aeruginosa* internalization requires more extensive and far ranging approaches. The use of RNA interference (RNAi) to rapidly and efficiently inhibit the expression of proteins [32] affords the possibility of carrying out unbiased forward genetic screens to identify host proteins critical to *P. aeruginosa* invasion. *Drosophila melanogaster* with its relatively small, non-redundant but evolutionarily conserved genome, provides an ideal “genetic” host in which to study host-pathogen interactions. Drosophila readily takes up double stranded RNA (dsRNA), allowing efficient inactivation of gene expression in whole flies as well as in Drosophila tissue culture cell lines. RNAi-based forward genetic screens in Drosophila S2 cells, a cell line derived from phagocytic hematopoietic cells [33], have been used successfully to identify new genes involved in cell division, phagocytosis, and recognition of bacteria [34]–[38].

In this study, we establish that *P. aeruginosa* infection of S2 cells mimics key aspects of mammalian cell infection including type III secreted effector-mediated modulation of bacterial entry, suggesting a conserved mode of entry. We used a library of dsRNAs representing conserved genes involved in the regulation of the actin cytoskeleton to systematically identify host genes required for *P. aeruginosa* uptake in Drosophila S2 cells. Our forward genetic screen revealed an invasion pathway for *P. aeruginosa* that involves Abl tyrosine kinase, its target Crk, the small GTPases Rac1, Cdc42, and p21-activated kinase (Pak1). We further validated the role of this signaling cascade in mammalian cells employing chemical, genetic, and siRNA-based approaches. This Abl-dependent pathway has not previously been associated with *P. aeruginosa* internalization and our studies reveal new complexities in the modulation of this pathway by the T3SS proteins ExoS and ExoT. Together our results demonstrate the potential of using RNAi-based screens to identify host molecules that are important in the pathogenesis of *P. aeruginosa* and that may serve as novel drug targets for treating infections resistant to conventional antibiotics.

## Results

### RNAi-based screen to identify host factors required for *P. aeruginosa* entry

**Figure 1:**
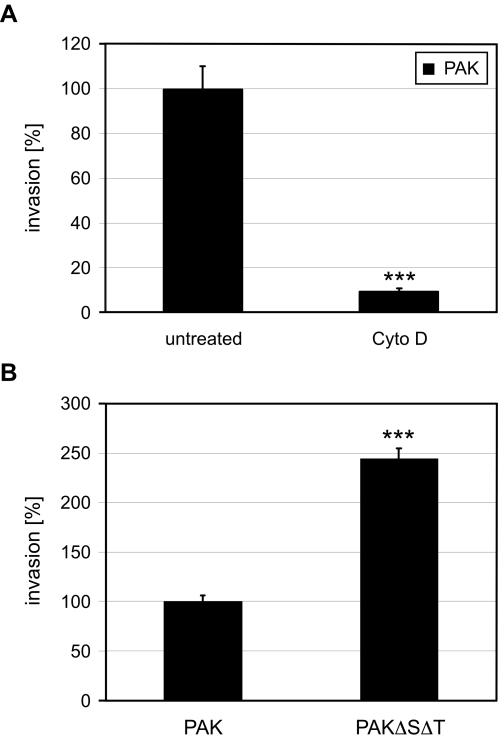
*P. aeruginosa* entry into Drosophila S2 cells mimics entry into mammalian cells. (A) Invasion of PAK into Drosophila S2 cells is actin-dependent. Drosophila S2 cells were treated with 10 µM Cytochalasin D and infected with PAK for 2 h at 28°C. The number of internalized bacteria was determined by performing an invasion assay. Results are normalized to untreated cells. ***p<0.001 compared to untreated cells. (B) The T3SS effector proteins ExoS and ExoT inhibit *P. aeruginosa* entry into S2 cells. Invasion of PAK and PAKΔSΔT into S2 cells after 2 h at 28°C were compared. The results were normalized with respect to invasion of PAK. ***p<0.001 compared to invasion of PAK into S2 cells.

To conduct a functional genomic screen to identify host cell factors required for internalization of *P. aeruginosa*, we exploited the susceptibility of Drosophila S2 cells, a macrophage-like cell line, to RNAi-mediated gene inactivation. Using a standard aminoglycoside protection assay to quantify bacterial internalization, we initially established that *P. aeruginosa* invasion of Drosophila S2 cells mimics entry into mammalian cells by assaying two important characteristics. First, as with mammalian cells, Cytochalasin D, an inhibitor of actin polymerization, diminished entry of *P. aeruginosa* strain K (PAK) into S2 cells (Figure 1A). PAK encodes the effector proteins ExoS, ExoT and ExoY, but lacks ExoU. Second, entry of PAKΔSΔT, an isogenic strain, in which the ExoS and ExoT genes have been deleted, into S2 cells was 2–8 fold more efficient than wild type PAK (Figure 1B); entry of PAKΔSΔT was also sensitive to cytochalasin D (data not shown). This finding is consistent with the known anti-internalization activity of ExoS and ExoT in mammalian cells [18],[24]. These results demonstrate that Drosophila S2 cells recapitulate important aspects of *P. aeruginosa* entry, including involvement of the actin cytoskeleton and translocation and functionality of the two T3SS effectors.

To identify host gene products crucial for the internalization of *P. aeruginosa*, we screened a library of dsRNAs representing phylogenetically conserved genes of *Drosophila melanogaster* that are known regulators of the actin cytoskeleton [37]. Given the known requirement for the actin cytoskeleton in *P. aeruginosa* invasion, we reasoned that this approach would yield a high likelihood of identifying host genes essential to *P. aeruginosa* invasion.

Drosophila S2 cells were treated with dsRNAs for 4 days and bacterial invasion assays with PAK were performed in triplicate three times. Invasion rates were normalized to S2 cell number for each dsRNA treatment to eliminate any apparent changes in invasion efficiency secondary to siRNA-mediated changes in cell number (Table S1 & S2). We chose to further study host proteins whose depletion reduced invasion by at least 33%, representing 36% of the dsRNAs tested (Table S1). For comparison, we also tested 23 random dsRNAs from a larger library and found that only 2 of 23 RNAs (9%) reduced entry (data not shown). These findings are consistent with our thesis that the subset of genes involved in regulating the actin cytoskeleton would be enriched for candidates involved in *P. aeruginosa* entry.

We predicted that depleting proteins known to directly affect actin assembly would inhibit *P. aeruginosa* invasion. Indeed, RNAi-mediated inactivation of Capping protein beta (Cpb), Kette, WASP, Sra-1, Abi, SCAR, and the p20 subunit of the Arp2/3 complex reduced invasion (Table S1). We also identified PI3K and Protein kinase B/Akt, kinases that we have previously shown to be required for PAK entry into mammalian cells [27]. The identification of host genes whose depletion is predicted or has already been shown to modulate internalization confirmed the validity of this methodology.

Interestingly, our screen identified several components of a signaling pathway that has not previously been implicated in *P. aeruginosa* invasion, including Abelson tyrosine kinase (Abl), its target CT10 regulator of Kinase (Crk), and p21-activated kinase (Pak). The identification of multiple components within a pathway indicated that this pathway might be relevant to uptake of *P. aeruginosa* in host cells. The identification of Abl kinase and its target protein Crk was even more intriguing as Crk had previously been shown to be targeted by the *P. aeruginosa* anti-internalization factor ExoT [15], although direct demonstration of the role of Crk in *P. aeruginosa* entry is lacking. We therefore further investigated the role of the Abl kinase pathway in the internalization of PAK into cultured mammalian epithelial cells using pharmacological, genetic, and biochemical approaches.

### Abl tyrosine kinase is required for invasion of *P. aeruginosa* in mammalian cells

**Figure 2:**
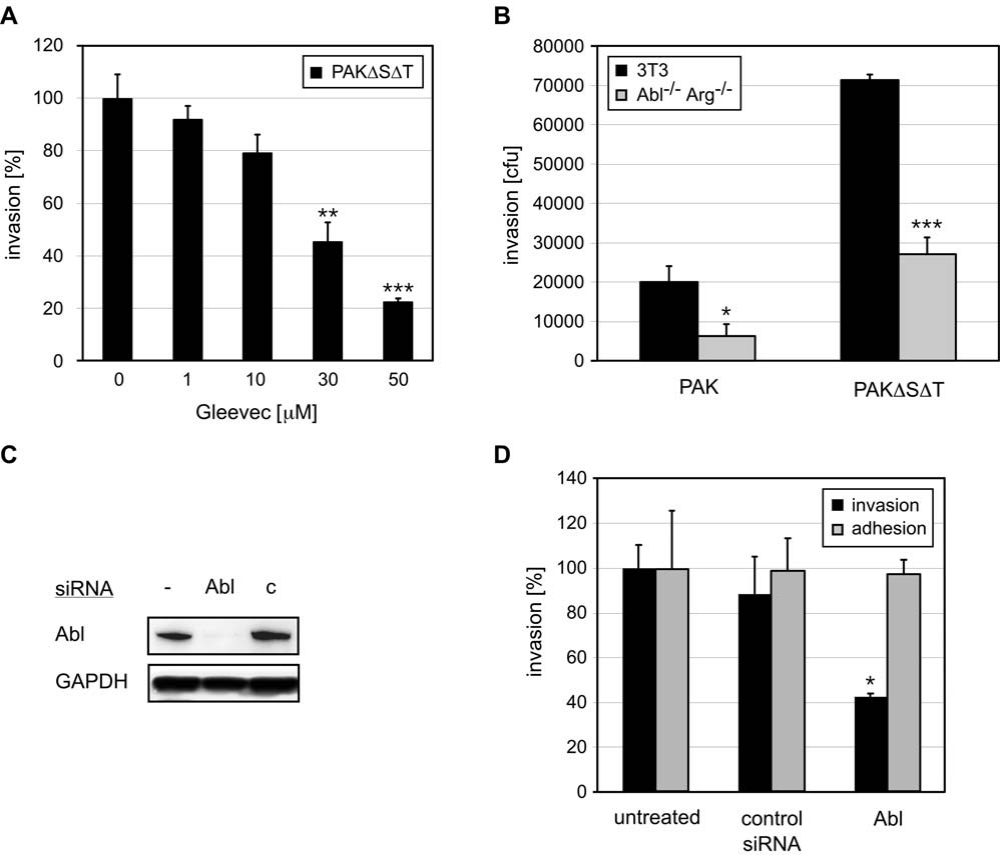
*P. aeruginosa* invasion into mammalian cells is dependent on Abl tyrosine kinases. (A) HeLa cells were infected with PAKΔSΔT for 1 h in the presence of the Abl inhibitor Gleevec (0–50 µM), and bacterial invasion was quantified. The results are normalized with respect to invasion in untreated cells. **p<0.01, ***p<0.001 compared to untreated cells. (B) Abl^−/−^Arg^−/−^ fibroblasts and parental 3T3 fibroblasts were infected with PAK or PAKΔSΔT for 1 h and bacterial uptake was measured by invasion assays. *p<0.05, ***p<0.001 compared to 3T3 fibroblasts. (C) HeLa cells were treated with Abl or control (c) siRNA. Cell lysates immunoblotted with an anti-Abl-antibody show decreased Abl protein levels compared to control siRNA-treated or untreated cells. GAPDH was used as loading control. (D) Standard invasion and adhesion assays were used to quantify the effect of Abl or control siRNA treatment on invasion and adhesion of PAKΔSΔT at 1 h into HeLa cells. The results are normalized with respect to invasion in untreated cells. *p<0.05 compared to control siRNA-treated cells.

The Abl family of non-receptor tyrosine kinases consists of two widely expressed members, Abl and Arg (Abl2) [39],[40]. Besides catalytic and protein-protein-interaction domains, Abl kinases contain a C-terminal actin-binding domain, a characteristic that is unique among all known tyrosine kinases. Abl kinases have been shown to regulate Rac1-dependent cytoskeletal dynamics that underlie protrusion formation in mammalian cells and have been implicated in the regulation of a number of cellular processes, including cell survival, proliferation, adhesion and motility [40]. Using Gleevec (STI571, imatinib), a well-characterized inhibitor of Abl tyrosine kinase activity [41],[42], we preliminarily assessed the role of Abl kinase in *P. aeruginosa* invasion into mammalian cells. Treatment with Gleevec inhibited PAK and PAKΔSΔT invasion into mammalian cells to the same extent in a dose-dependent manner without affecting bacterial adhesion, or host or bacterial viability (Figure 2A and data not shown). Gleevec did not inhibit *Salmonella typhimurium* invasion or adhesion in HeLa cells (Figure S1A).

As Gleevec is not entirely specific for Abl/Arg kinases [43],[44], we confirmed these results by quantifying the entry of PAK and PAKΔSΔT into 3T3 fibroblasts derived from mice lacking both Abl and Abl-related kinase Arg [39]. Consistent with the known effects of ExoS and ExoT on bacterial entry into mammalian cells [18],[24], internalization of PAK into 3T3 fibroblasts was about 3.5-fold less efficient than internalization of PAKΔSΔT. Furthermore, entry of either strain in the Abl/Arg deficient cells was decreased to 60–70% compared to entry into parental cells (Figure 2B). The absence of Abl kinases did not have an effect on bacterial binding (data not shown). Entry of *S. typhimurium* into Abl/Arg depleted cells was unaffected (Figure S1B). Finally, we demonstrated that siRNA-mediated depletion of Abl (Figure 2C) decreased invasion of PAK (data not shown) and PAKΔSΔT approximately 2-fold compared to untreated and control siRNA-exposed cells (Figure 2D), but did not affect adhesion (data not shown). Collectively, these results indicate that efficient invasion of *P. aeruginosa* into mammalian cells requires Abl tyrosine kinase activity.

### Crk is required for *P. aeruginosa* invasion

Crk is an SH2- and SH3-domain containing adaptor protein which has been shown to be involved in multiple cellular processes including phagocytosis, cell adhesion, cell migration, and immune responses [45]. CrkI and CrkII are splicing variants that differ by the presence of an additional C-terminal SH3 domain in CrkII and a tyrosine residue between the two SH3 domains. CrkII is phosphorylated by Abl kinase at tyrosine 221, resulting in a conformational change that affects its subcellular localization and alters its ability to interact with other signaling effectors [46]. CrkI and II have also been shown to be the major targets of the ADPRT domain of the effector protein ExoT. ADP-ribosylation of arginine 20 of the SH2 domain of Crk by ExoT disrupts the interaction of this domain with binding partners [47]. However, a direct role for Crk in *P. aeruginosa* entry has not previously been demonstrated.

**Figure 3:**
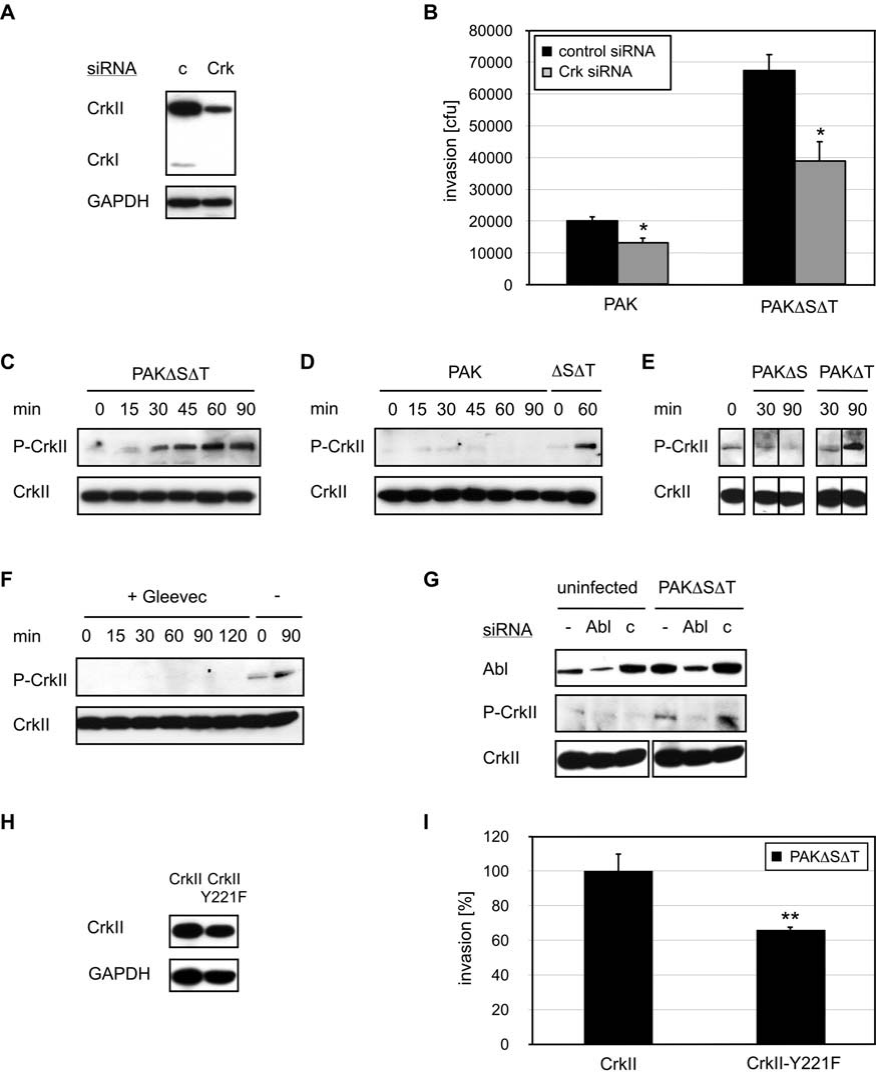
Abl-dependent Crk phosphorylation is required for *P. aeruginosa* invasion. (A) HeLa cells were treated with Crk or control (c) siRNA and cell lysates were immunoblotted with an anti-CrkI/II-antibody. GAPDH was used as loading control. (B) Standard invasion assays were used to quantify the effect of Crk or control siRNA treatment of invasion of PAK or PAKΔSΔT at 1 h into HeLa cells. *p<0.05 compared to control RNA-treated cells. (C–E) HeLa cells were infected with the indicated strains for the indicated times, lysed and immunoblotted with anti-phospho-CrkII-Y221 and anti-CrkI/II. (F, G) HeLa cells were infected with PAKΔSΔT for indicated times in the presence of 30 µM Gleevec (F) or after treatment with Abl or control siRNA (G) and cell lysates were immunoblotted with anti-phospho-CrkII-Y221 and anti-CrkI/II. (H) HeLa cells were transfected with pCAGGS-CrkII and pCAGGS–CrkII-Y221F for 16 h and cell lysates were immunoblotted with an anti-CrkI/II antibody. GAPDH was used as loading control. (I) Standard invasion assays were used to quantify the effect of over-expression of CrkII or CrkII-Y221F on invasion of PAKΔSΔT at 1 h into HeLa cells. **p<0.01 compared to cells over-expressing wild type CrkII.

Using RNAi, we tested whether Crk plays a role in *P. aeruginosa* entry into mammalian cells. Following depletion of CrkI and CrkII by dsRNA directed against both isoforms (Figure 3A), invasion of PAK and PAKΔSΔT was reduced to 70±11% and 58±9%, respectively, compared to bacterial uptake in control RNAi-treated cells (Figure 3B). CrkI/II depletion had no effect on adhesion of PAK to host cells (data not shown) or on internalization of *S. typhimurium* (Figure S2). Since Crk is a known target of ExoT, it might have been expected that depletion of Crk would affect invasion of PAKΔSΔT to a greater extent than invasion of the ExoT-expressing wild type strain. Our finding that invasion of both strains was decreased to similar extents may be explained by the observation that the effects of the translocated effector proteins are only apparent after a delay (see below). The implication of these results will be discussed later in more detail. Altogether, our finding that depletion of Crk decreased PAK invasion into Drosophila S2 cells as well as into mammalian epithelial cells indicates that Crk is required for *P. aeruginosa* invasion and is consistent with the notion that ExoT inhibits internalization at least in part by disrupting Crk function.

### *P. aeruginosa* induced phosphorylation of CrkII is Abl-dependent and is required for bacterial internalization

Having shown that Abl kinases and Crk are required for internalization, we tested Abl kinase activation by assaying CrkII phosphorylation in response to bacterial infection. Lysates of HeLa cells infected with PAK and PAKΔSΔT were immunoblotted with antibodies that specifically recognize total Crk or CrkII phosphorylated on tyrosine 221 (Figures 3C and D). For both PAK and PAKΔSΔT, increased CrkII phosphorylation could be detected as early as 15 minutes post infection. In the case of PAKΔSΔT, CrkII phosphorylation increased over time and remained readily detectable up to 90 minutes post infection (Figure 3C). In contrast, the fraction of CrkII phosphorylation in PAK-infected cells did not further increase after 15 minutes and was undetectable by 60 minutes post infection (Figure 3D). These results suggest that upon binding, *P. aeruginosa* activates Abl, which leads to CrkII phosphorylation. Subsequent T3SS-dependent translocation of ExoS and/or ExoT by the wild type strain PAK inhibits further CrkII phosphorylation. While both effector proteins exhibit GAP activity towards Rac1 and Cdc42 [7],[8],[48], signaling molecules that are likely downstream of Crk, ExoT is known to directly interfere with Crk function. Indeed, infection with isogenic PAK mutants lacking either ExoT or ExoS revealed that ExoT was responsible for the inhibition of PAKΔSΔT-induced CrkII phosphorylation (Figure 3E).

To further test whether Abl is responsible for the phosphorylation of CrkII upon infection with *P. aeruginosa*, HeLa cells were either treated with the Abl kinase inhibitor Gleevec (Figure 3F) or depleted of Abl by siRNA (Figure 3G). Either treatment abrogated PAKΔSΔT-induced phosphorylation of CrkII, indicating that Abl is required for the PAKΔSΔT induced phosphorylation of CrkII.

We determined whether phosphorylation of CrkII at tyrosine 221 is required for internalization of PAK by examining the effect of over-expression of either wild type CrkII or a non-phosphorylatable CrkII mutant (CrkII-Y221F; [49]) in HeLa cells on bacterial internalization. Each protein was over-expressed to similar levels (Figure 3H). As shown in Figure 3I, over-expression of CrkII-Y221F resulted in a 34% reduction of the invasion rate of PAKΔSΔT compared to invasion in HeLa cells over-expressing wild type CrkII. Expression of CrkII-Y221F in HeLa cells would not be expected to completely abolish *P. aeruginosa* internalization as these cells still express endogenous CrkII. Nonetheless, these data demonstrate that phosphorylation of CrkII by Abl kinase is important for efficient *P. aeruginosa* internalization.

### Rac1 and Cdc42 contribute to internalization of *P. aeruginosa*

**Figure 4:**
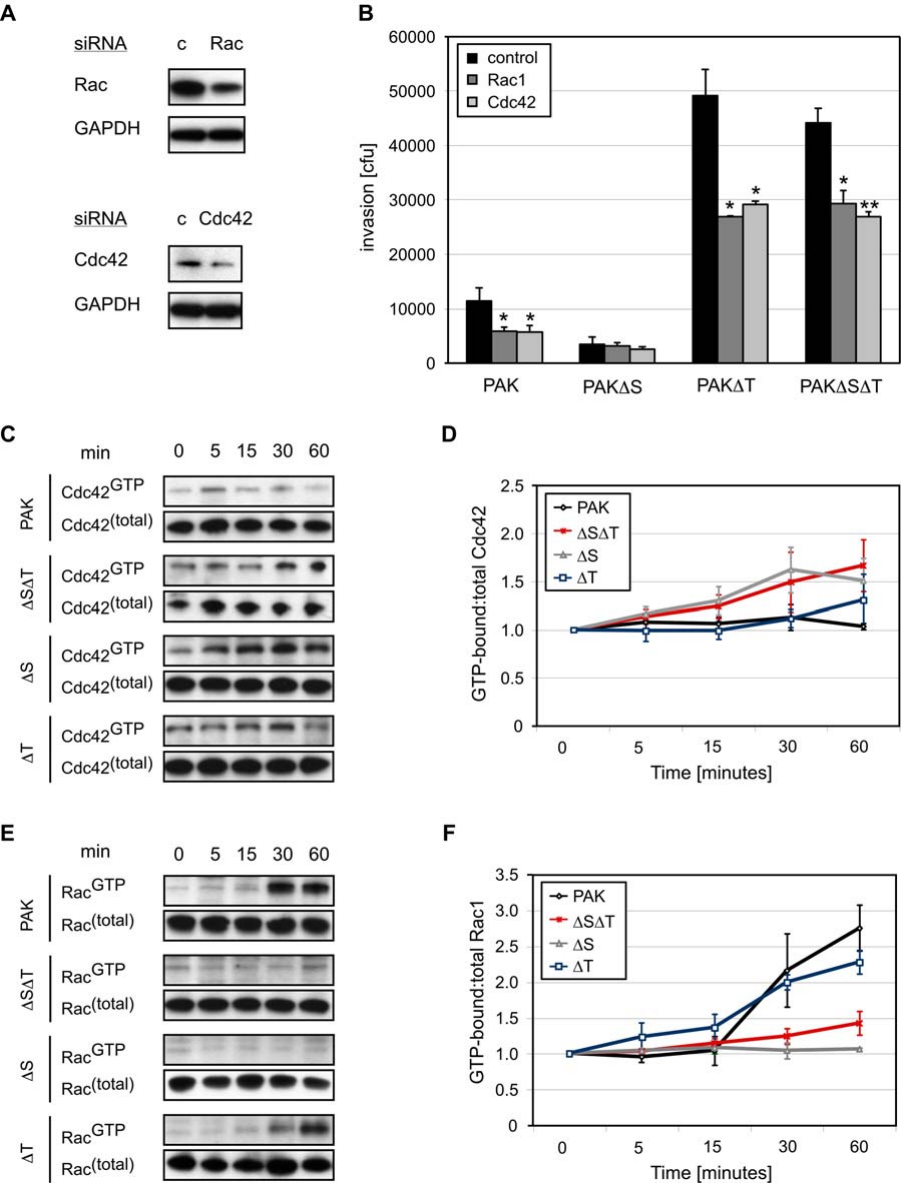
Rac1 and Cdc42 are required for invasion of *P. aeruginosa* into mammalian cells. (A) HeLa cells were treated with Rac1, Cdc42 or control (c) siRNA. Cell lysates were immunoblotted with anti-Rac1 and anti-Cdc42, respectively. GAPDH was used as loading control. (B) Internalization of PAK, PAKΔS, PAKΔT and PAKΔSΔT at 1 h was quantified in HeLa cells treated with control siRNA or siRNA against Rac1 or Cdc42. *p<0.05, **p<0.01 compared to control RNA-treated cells for each bacterial strain. (C–F) GTPase activation assays. HeLa cells were infected with PAK, PAKΔSΔT, PAKΔS and PAKΔT for the indicated times. Lysates were incubated with Pak1-PBD to precipitate GTP-bound Cdc42 and GTP-bound Rac1. The bound proteins and cell lysates were examined by immunoblotting with anti-Cdc42 (C) and anti-Rac1 (E), respectively. The experiments were performed 3 times and a typical gel is shown. (D, F) The GTPases activation assays to assess Cdc42 (D) or Rac1 (F) activation upon infection with PAK, PAKΔSΔT, PAKΔS and PAKΔT were quantified by densitometry. Shown are the mean values +/- SD from three independent experiments.

The Rho family GTPases have previously been linked to Abl through genetic studies in Drosophila and loss-of-function studies in mammalian cells [40],[50]. In addition, Rac1- dependent signaling has been shown to be regulated by CrkII, whose ability to interact with other signaling molecules is modulated upon phosphorylation [49]. Previous studies demonstrated that Rho-family GTPase activity is required for internalization of a different strain of *P. aeruginosa,* PA103 [25]. Although our initial RNAi screen suggested only minor effects of Rac1 and Cdc42 on the entry of PAK into S2 cells (Table S1), RNAi mediated depletion of either GTPases (Figure 4A) inhibited internalization of PAK into mammalian cells (Figure 4B). We further tested the effect of Rac1 and Cdc42 depletion on PAKΔSΔT, PAΔS and PAKΔT. Entry of PAK, PAKΔSΔT and PAKΔT in Rac1-depleted HeLa cells was diminished to 61±8%, 67±5% and 55±1% (Figure 4B), respectively, compared to bacterial entry in control-siRNA treated cells, while entry of PAKΔS was unaffected (90±18%). Depletion of Cdc42 in HeLa cells decreased the entry of PAK, PAKΔSΔT, PAKΔS and PAKΔT to 60±13%, 62±2%, 71±12% and 59±1%, respectively (Figure 4B). Bacterial binding was unaffected (data not shown).

**Figure 5:**
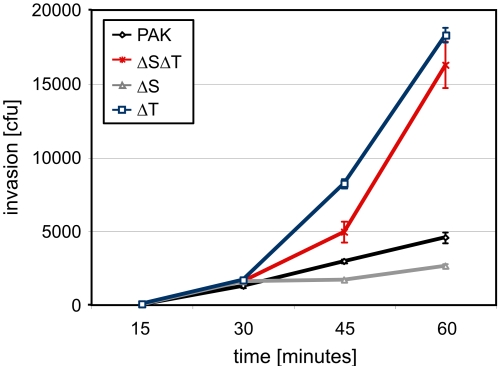
Kinetics of internalization of PAK into mammalian cells. Invasion of PAK, PAKΔSΔT, PAKΔS and PAKΔT in HeLa cells at the indicated times was quantified. Shown are the mean +/− SD from three independent experiments.

We further examined in detail the kinetics of PAK internalization into epithelial cells. Figure 5 reveals that all four strains (PAK, PAKΔSΔT, PAKΔT, PAKΔS) are equally invasive at early times of infection, providing a potential explanation for why depletion of host cell targets of ExoS and ExoT similarly reduced invasion of PAK and PAKΔSΔT. The effects of the effector proteins ExoS and ExoT are only apparent at 30 minutes post infection. This delay correlates with the kinetics of translocation of the effector proteins into the host cell cytosol (P. Balachandran, personal communication). After 30 minutes of infection, only a limited further increase in the entry of PAK or PAKΔS was observed, suggesting that the anti-internalization activities of ExoT prevailed. By 1 h post infection, there were 4-fold more intracellular PAKΔSΔT than the wild type strain (Figure 5). Interestingly, PAKΔT, the strain that expresses only ExoS, was even slightly more invasive than PAKΔSΔT.

We next looked for a correlation between the invasion time course and activation of Rac1 and Cdc42. ExoS and ExoT are predicted to have complicated and even opposing effects on Rac1 and Cdc42 activation: both ExoS and ExoT harbor GAP activity towards Rho, Rac1, and Cdc42 [16], but ADP ribosylation of Rac1 by ExoS has also been shown to lead to Rac1 activation in some cell types [51]. Direct correlation of invasion and activation of these proteins is further complicated by virtue of the fact that we are examining activation of total cellular Rac1 or Cdc42 whereas the relevant effect in the host cell could be due to local changes in concentration or activation. Moreover, as described in the preceding section, ExoT affects other targets (e.g. CrkII), which will also impact the overall invasion rate. Nevertheless, we measured the fraction of activated Rac1 and Cdc42 at various times after infection with PAK, PAKΔSΔT, PAKΔS or PAKΔT.

Between 30 minutes and 1 hour, the time at which the strains began to show divergent invasion profiles, a slight activation of Cdc42 is apparent in PAKΔS, PAKΔT and PAKΔSΔT relative to PAK (Figures 4C and D). This finding would be consistent with both ExoS and ExoT contributing to Cdc42 inhibition through their respective GAP activities. During this 30 minutes to 1 hour time frame ExoS apparently promotes activation of Rac1, as PAK and PAKΔT exhibit Rac1 activation, while PAKΔS and PAKΔSΔT do not (Figures 4E and F).

The following model may account for the observed requirement for Cdc42 and Rac1 along with the complex changes in total cellular Cdc42 and Rac1 activation observed over the first hour of invasion. We propose that PAKΔSΔT causes local activation and/or recruitment of Rac1 and Cdc42, resulting in entry into non-phagocytic cells. PAKΔT is reproducibly slightly more invasive than PAKΔSΔT at later time points, likely due to the ExoS-mediated Rac1 activation. Both strains lead to phosphorylation of CrkII, which, as we demonstrated above, also contributes to invasion of these strains. PAK, though it shows similar (and also ExoS-dependent) Rac1 activation relative to PAKΔT, is less invasive, presumably due to ExoT-mediated inhibition of Cdc42 and the ExoT-mediated inhibition of CrkII phosphorylation. Our observation that PAK activates Rac1 further suggests that the ADPRT activity of ExoS prevails over the GAP activity of ExoT. PAKΔS does not express ExoS and can therefore not activate Rac1. In addition, PAKΔS is subject to ExoT-mediated inhibition of Rac1 and Cdc42 (compared to PAKΔSΔT) as well as ExoT-mediated inhibition of CrkII phosphorylation. Consequently this strain shows even less invasion than PAK.

### Pak1 is required for *P. aeruginosa* entry into mammalian cells

**Figure 6:**
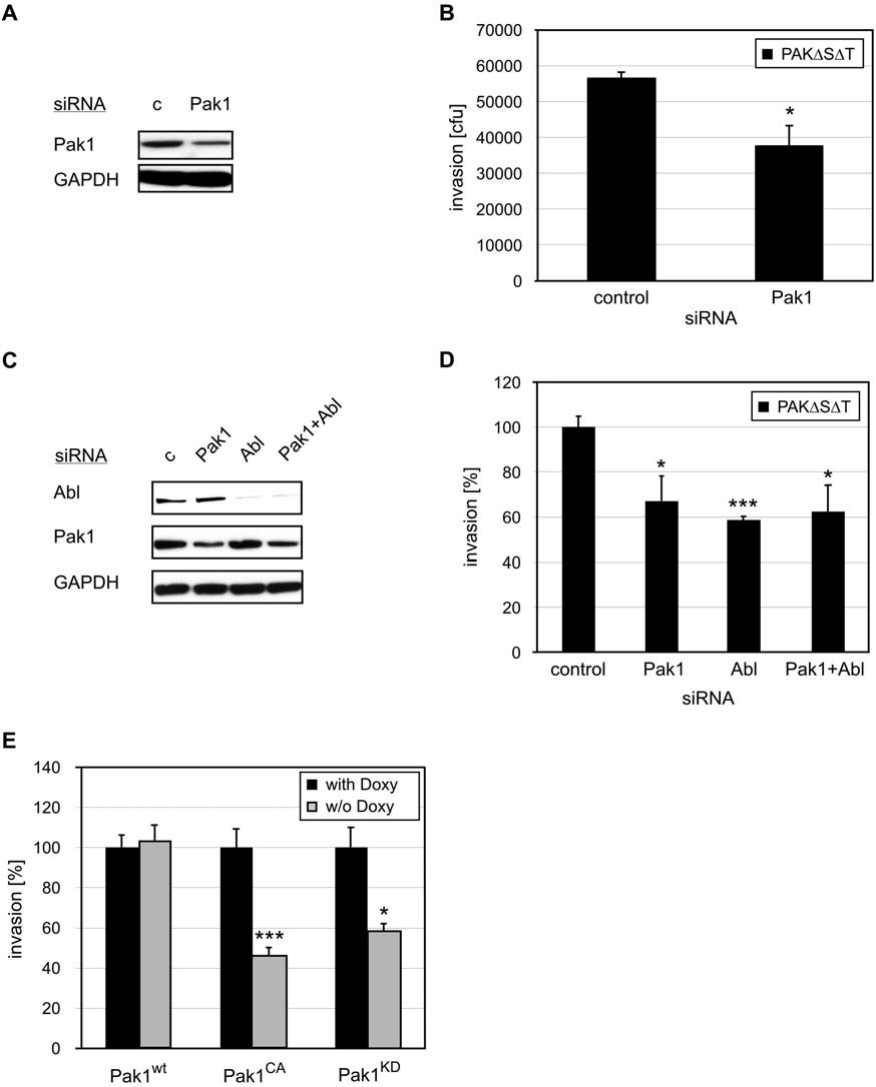
Invasion of *P. aeruginosa* is Pak1 dependent. (A) HeLa cells were treated with Pak1 or control (c) siRNA. Cell lysates were immunoblotted with anti-Pak1. GAPDH was used as loading control. (B) Internalization of PAKΔSΔT at 1 h was quantified in HeLa cells treated with control siRNA or siRNA against Pak1. *p<0.05 compared to control siRNA-treated cells. (C) HeLa cells were treated with Pak1 and/or Abl siRNA or control siRNA. Cell lysates immunoblotted with anti-Pak1 and anti-Abl. GAPDH was used as loading control. (D) Internalization of PAKΔSΔT at 1 h was quantified in HeLa cells treated with control siRNA or siRNA against Pak1, Abl, and Pak1+Abl. Results are normalized to control siRNA-treated HeLa cells. *p<0.05, ***p<0.001 compared to control siRNA-treated cells. (E) MDCK cells were induced for 18 h to express human Pak1 (Pak1^wt^), a dominant-negative (Pak1^DN^) or a constitutively active (Pak1^CA^) mutant of Pak1 by removal of doxycycline. Invasion of PAKΔSΔT at 1 h was quantified and the results were normalized with respect to uninduced cells. *p<0.05, ***p<0.001 compared to uninduced cells.

Pak1 belongs to a family of serine/threonine kinases and becomes strongly activated upon binding of activated Rac1 and Cdc42 to their GTPase binding domain (PBD). Pak1 also plays a role in growth arrest upon wound closure. Interestingly, this function is dependent upon the ability of Pak1 and its guanine exchange factor (GEF) Pix to localize to focal contacts and is disrupted in both dominant negative and constitutively active mutants [52]. As shown in Figure 6B, siRNA-mediated depletion of Pak1 in HeLa cells (Figure 6A) decreased PAKΔSΔT invasion approximately 2-fold. Bacterial adhesion was not affected (data not shown). In addition, simultaneous depletion of Pak1 and Abl (Figure 6C) did not additively inhibit *P. aeruginosa* invasion (Figure 6D), suggesting that Abl kinase and Pak1 function in the same pathway in *P. aeruginosa* invasion.

We confirmed these results using MDCK cells that can be induced to express human Pak1, a kinase-dead mutant of human Pak1 (Pak1^KD^; K299R) or a constitutively active Pak1 allele (Pak1^CA^; T423E). Figure 6E demonstrates that over-expression of either the kinase-dead or the constitutively active form of Pak1 inhibits PAKΔSΔT invasion. The results are consistent with published reports showing that cycling of Pak1 between its active and inactive form is critical for its function [52].

## Discussion

Understanding how pathogens subvert the host cell cytoskeleton to induce their own internalization is of great interest, opening new avenues to develop treatments to control antibiotic-resistant infections as well as furthering our understanding of fundamental aspects of cell biology. In the experiments reported here, we used RNAi-mediated gene inactivation in Drosophila S2 cells to carry out an unbiased forward genetic screen to identify host molecules crucial to entry of *P. aeruginosa*. As S2 cells are phagocytic in origin, our screen had the potential to identify genes involved in phagocytosis or in pathogen-directed uptake into non-phagocytic cells. We identified the tyrosine kinase Abl, the adaptor protein Crk, the Rho family GTPases Rac1 and Cdc42, and Pak as components of a host signaling pathway which has not previously been demonstrated to be required for *P. aeruginosa* entry. Using comprehensive and complementary approaches, we validated the role of the Abl kinase pathway in *P. aeruginosa* entry into mammalian epithelial cells. Remarkably, three of its components, Crk, Rac1 and Cdc42, are known targets of ExoS and/or ExoT, T3SS effector proteins of *P. aeruginosa* that have been shown to modulate *P. aeruginosa* internalization into mammalian cells [18],[24]. Our results further reveal new complexities in the regulation of bacterial entry by ExoS and ExoT.

Through the use of a chemical inhibitor of Abl kinase, an Abl/Arg deficient cell line, and RNAi-mediated depletion of Abl, we demonstrate that this cytoplasmic tyrosine kinase is essential for efficient internalization of *P. aeruginosa* by mammalian cells (Figure 2). Abl kinase has been shown to be a key component of various steps in the infection of several pathogens, including actin motility in poxvirus infection, pedestal formation in enterophathogenic *E. coli*, and the entry of Coxsackievirus, and *Shigella flexneri* into non-phagocytic cells [50], [53]–[55]. However, the requirement for Abl in *P. aeruginosa* internalization does not simply reflect utilization of a general phagocytic pathway, as Abl is not required for the phagocytosis of dead bacteria [36]. Likewise, Abl depletion apparently does not affect the uptake of several other pathogens, including *S. typhimurium* (Figure S1), *Listeria monocytogenes*, *Mycobacterium fortuitum*, and *Candida albicans* [34],[35],[38]. Taken together, these results suggest that a subset of microbial pathogens subvert Abl-dependent pathways during pathogenesis.

Our data also provide new evidence that Crk plays a role in *P. aeruginosa* internalization (Figure 3B), that CrkII is phosphorylated by Abl upon *P. aeruginosa* infection (Figures 3C-G), and that the phosphorylation of CrkII contributes to the internalization of *P. aeruginosa* (Figure 3I). The phosphorylation of CrkII at tyrosine 221, which is required for its membrane localization, has been shown to modulate the ability of this adaptor protein to interact with other signaling molecules and to regulate the localization of Rac1 and Rac1-dependent signaling [49]. Phosphorylation of CrkII has also been demonstrated to be essential for Rac1 and Cdc42 activation upon *Shigella* infection [50]. Based on these findings, we postulate that infection with *P. aeruginosa* leads to phosphorylation of CrkII, facilitating its transport to the plasma membrane, where it interacts with other signaling molecules such as the small GTPases, eventually leading to bacterial internalization. The role of Crk in *P. aeruginosa* internalization is even more intriguing as this adaptor protein has been identified as the substrate for the T3SS effector ExoT [15]. ExoT has been shown to ADP ribosylate Crk on arginine 20 of its SH2 domain, disrupting its ability to interact with Paxillin and p130Cas [47]. Our data also suggest that ExoT inhibits CrkII phosphorylation (Figures 3C–E). Thus, upon translocation of its effector protein ExoT, *P. aeruginosa* can downregulate its internalization, at least in part by disruption of CrkII phosphorylation and function.

This study further reveals that invasion of PAK into epithelial cells is at least in part a Cdc42 and Rac1 dependent process (Figure 4B) that is subject to complex regulation. Using isogenic mutants in ExoS and/or ExoT, we examined the state of Rac1 and Cdc42 activation, the effect of depletion of Rac1 or Cdc42, and the kinetics of entry to formulate the following model. The effector deficient strain, PAKΔSΔT, likely locally activates Rac1 and Cdc42 to enhance entry into non-phagocytic cells, possibly through the insertion of the T3SS complex. PAKΔT, which translocates ExoS, is even more invasive than PAKΔSΔT, likely because of enhanced activation of Rac1 by the ADPRT activity of ExoS. Our finding that depletion of Rac1 or Cdc42 diminished entry suggests that there may be local activation of Cdc42 in addition to the observed ExoS-dependent activation of Rac1. PAKΔS is the least invasive of the four strains, and is least affected by depletion of Rac1 or Cdc42. This finding suggests that ExoT is able to effective abrogate Rac1 and Cdc42 activation. Finally, the phenotype of PAK may be explained as a complex combination of the synergistic and antagonistic effects of ExoS and ExoT. It is less invasive than PAKΔSΔT and PAKΔT, likely because the GAP activity of ExoT partially counteracts the activation of Rac1 by ExoS.

Previously, our lab reported that ectopic expression of ExoS in PA103ΔUΔT, a *P. aeruginosa* strain that does not normally express ExoS, inhibited internalization into macrophages but variably inhibited internalization into MDCK cells [18]. These disparate results are readily explained by reports showing that the ability of the ADPRT domain of ExoS to activate Rac1 are cell type specific; Rac1 activation was observed in fibroblasts and epithelial cells, but not macrophages [51],[56]. Our finding that ExoS has a slight stimulating effect on bacterial internalization into epithelial cells is particularly remarkable as it might represent a mechanism that explains why ExoS-expressing strains of *P. aeruginosa* are more invasive than strains that do not express ExoS. Furthermore, it implies that the effect of ExoS on invasion is context (i.e. cell type) specific. The exact physiological consequence of this remains to be determined, but it is striking that the vast majority of *P. aeruginosa* strains produce both ExoS and ExoT [57]. It is interesting to speculate that this imparts a flexibility that allows PAK to enter epithelial cells, such as those that line the mucosal barrier, while avoiding uptake by macrophages. Alternatively or in addition, ExoS/ExoT producing strains may exhibit enhanced fitness in the environment.

Our work further demonstrates a role for Pak1 in *P. aeruginosa* invasion. Pak1 belongs to a family of highly conserved serine/threonine kinases that are implicated in cytoskeletal rearrangements induced by GTP-bound forms of Rac1 and Cdc42 [58] (Figure 6). Interestingly, expression of a constitutively active mutant as well as a kinase-dead mutant of Pak1 inhibited bacterial internalization (Figure 6E). These findings corroborate that cycling of this kinase between an active and inactive state is required for its function [52].

Pak1 may facilitate *P. aeruginosa* invasion by regulating Arp2/3-dependent actin polymerization. Indeed, the Arp2/3 complex is also required in *P. aeruginosa* invasion (Table S1). Pak1 has been shown to interact both *in vivo* and *in vitro* with p41-Arc, a putative regulatory component of the human Arp2/3 complex [59]. Pak1 phosphorylation of p41-Arc regulates its localization with the Arp2/3 complex in the cortical nucleation regions of cells [59]. This interaction may represent a mechanism by which the signaling cascade triggered by *P. aeruginosa* influences the function of the Arp2/3-complex, leading to the formation of new actin filaments and lamellipodia, and eventually to bacterial uptake.

The activation of the Arp2/3 complex is also mediated by the Wiscott-Aldrich syndrome proteins WASP and WAVE, which are known effectors of Cdc42 and Rac1, respectively [60],[61]. As RNAi mediated depletion of WASP and WAVE decreased internalization of *P. aeruginosa* into S2 cells (Table S1), these proteins may also be involved in processes leading to bacterial uptake. This is furthermore supported by the finding that depletion of Abi, Sra-1 and Kette, which form a complex that regulates the function of WASP and WAVE in coordinating the formation of F-actin [61], also affected bacterial internalization (Table S1). Whether these observations are relevant to non-phagocytic cells remains to be determined.

Our RNAi screen also identified Phosphatidylinositol 3-kinase (PI3K) and Protein kinase B/Akt as host molecules that contribute to efficient *P. aeruginosa* internalization. Indeed, recent work in our laboratory demonstrated that PI3K and its downstream effector Protein kinase B/Akt are required for internalization of PAK in MDCK cells [27]. It will be important to determine if the PI3K/Akt pathway intersects with the Abl kinase internalization pathway. Preliminary results using the pharmacological Abl inhibitor Gleevec and the PI3K inhibitor LY294002 suggest that these signaling pathways may be separate (Pielage and Engel, unpublished data). It is also possible that the interaction between these two pathways occurs further downstream, such as at the level of the Rho family GTPases. Alternatively, they may share a mutual receptor, though further work will be required to elucidate the details.

As clinically important antibiotic resistance of *P. aeruginosa* continues to increase, the identification of host genes essential for the pathogenesis of *P. aeruginosa* infections may lead to new drug targets. The Abl inhibitor Gleevec, a well tolerated drug which has become a mainstay for the treatment for chronic myelogenous leukemia and stromal tumors with few side effects [41], has been shown to protect against vaccinia virus infection in mice [53] and may prove to be effective against *P. aeruginosa* and other pathogens that subvert Abl kinase-dependent pathways. As drugs such as Gleevec affect host instead of bacterial proteins, they are much less likely to engender resistance compared to conventional antimicrobial treatments, and may be applicable to a wide range of pathogens. Future studies will be directed towards assessing these host cell targets as candidates for new therapies.

## Materials and Methods

### Bacterial strains and cell lines

*P. aeruginosa* strain K (PAK), PAKΔS (ExoS::omega), PAKΔT (ExoT::gent) and PAKΔSΔT (ExoS::omega, ΔT::gent) [23] were routinely grown with vigorous aeration overnight in low salt (90 mM NaCl) Luria-Bertani (LB) broth at 37°C. Overnight cultures were diluted 1∶30 in LB, grown to a mid-log OD_600_ and adjusted to OD_600_ of 0.1 in cell culture medium.

*Salmonella typhimurium* SL1344 (obtained from Dr. S. Falkow, Stanford) was grown overnight without shaking in high salt (180 mM NaCl) LB broth. Overnight cultures were diluted 1∶20 in cell culture medium and grown to an OD_600_ of 0.1.

S2 cells (obtained from Dr. R. Vale, UCSF) were cultured in Schneider's Drosophila medium (Invitrogen) supplemented with 10% heat-inactivated fetal bovine serum (FBS; HyClone) at 28°C. HeLa cells (ATCC CCL-2) were routinely grown in minimal essential medium (MEM, UCSF Cell Culture Facility) supplemented with 10% heat-inactivated FBS. 3T3 cells or 3T3 cells derived from Abl^−/−^Arg^−/−^ mice [39] were grown in Dulbecco's minimal essential medium (DMEM; UCSF Cell Culture Facility) supplemented with 20% heat-inactivated FBS. MDCK cells expressing human wild type Pak1, a constitutively active allele (Pak1^CA^; T423E) or a kinase-dead allele (Pak1^KD^; K299R) under control of a controllable transactivator using the tet-off system [52] were cultured in DMEM containing 5% FBS and 20 ng/ml doxycycline (Sigma). To induce expression of the transgene, cells were grown in the absence of doxycycline. All mammalian cells were maintained at 37°C in a humidified atmosphere containing 5% CO_2_.

### Adhesion and invasion assays

Invasion and adhesion assays were performed as described previously [62] with minor modifications. 1×10^6^ Drosophila S2 cells were seeded into 24-well plates and infected with PAK growing in exponential phase (multiplicity of infection (MOI) of 30) for 2 h at 28°C. Alternatively, 1×10^5^ HeLa cells were seeded in 24-well plates and incubated overnight. The next day, cells were infected with exponentially growing bacteria (MOI of 30) for 1 h (except where noted) at 37°C. For assays performed in the presence of inhibitors, cells were pre-incubated with medium containing Cytochalasin D from *Zygosporium mansonii* (inhibitor of actin polymerization; 10 µM final concentration; Sigma) or Gleevec (STI571; selective inhibitor of Abl tyrosine kinase) [41] at 37°C and 5% CO_2_ for 1 h prior to the infection. All invasion and adhesion assays were done in triplicate and error bars indicate standard errors of the mean (SEM). p values were calculated using the two-tailed student's t test.

### Transient transfection

4×10^4^ HeLa cells were seeded per well of a 24-well-plate. 24 h later, cells were transfected with pCAGGS-CrkII (wild type Crk II), pCAGGS-CrkII-Y221F (non-phosphorylatable CrkII mutant) [49] and pCAGGS (vector only) using Effectene (Qiagen, Valencia, CA) following the manufacturer's instructions. After an incubation period of 16 h, invasion assays were performed and cells were lysed to check for efficacy of transfection.

### RNAi-mediated gene inactivation in Drosophila S2 cells

dsRNAs were generated from a library of DNA templates for 77 genes encoding actin-binding proteins [37] by *in vitro* transcription reactions for 6 h at 37°C using RiboMAX™ Large Scale RNA production system T7 (Promega). 5×10^4 ^S2 cells were seeded into 96-well-plates, incubated with a final concentration of 10 µg/ml dsRNA for 4 days and infected with *P. aeruginosa* (MOI of 30) for 2 h at 28°C following the protocol described above.

### Short interfering RNA (siRNA)-mediated protein depletion

siRNAs were purchased from Santa Cruz Biotechnology: Abl (sc-29843), CrkII (sc-37072), Cdc42 (sc-29256), Rac1 (sc-36351), Pak1 (sc-29700) and control siRNA (sc-37007). HeLa cells were transfected with siRNAs according to the manufacturer's instructions. After 42 h, standard adhesion and invasion assays were performed. In parallel, lysates were immunoblotted with appropriate antibodies to evaluate the efficiency of protein depletion.

### Preparation of cell lysates and immunoblotting

2×10^6^ HeLa cells were seeded onto 10 cm plates, serum-starved over night, and infected with *P. aeruginosa* (MOI of 100) for the indicated times. Cells were washed with PBS and lysed in 1% Triton X-100 in PBS supplemented with proteinase inhibitors (Complete; Roche Diagnostics) for 20 minutes at 4°C. Cell lysates were clarified by centrifugation and separated by SDS-PAGE. After transfer to PVDF membranes (Immobilon, Millipore), membranes were blocked in 5% milk in PBS-T (PBS supplemented with 0.1% Tween 20), incubated with primary antibodies overnight at 4°C, washed in TBS-T buffer (20 mM Tris-HCl, pH8, 137 mM NaCl, 0.7% Tween 20), incubated with appropriate horseradish peroxidase-conjugated secondary antibodies for 1 h at RT, washed again and developed using a chemiluminescence kit (ECL, Amersham Pharmacia).

### GTPase activation assay

2×10^6^ HeLa cells were seeded onto 10 cm plates and serum-starved overnight. The next day, cells were infected with *P. aeruginosa* (MOI of 100) for the indicated times. To precipitate GTP-bound Rac1 and Cdc42, cell lysates were incubated with Pak1-PBD agarose (Rac1/Cdc42 activation assay, Upstate Biotechnology) following the manufacturer's instructions. Samples were run on 12% Bis-Tris gels and immunoblotted as described above. For quantification GTP-bound Rac1 or Cdc42 was compared to total Rac1 or Cdc42 and normalized to uninfected cells.

### Antibodies

Antibodies include mouse-anti-c-Abl (sc-23, Santa Cruz Biotechnology; 1∶400), mouse-anti-Crk (BD Transduction Laboratories; 1∶2,500), rabbit-anti-phospho-CrkII (Tyr221; Cell Signaling; 1∶500), rabbit-anti-Pak1 (N-20; sc-882, Santa Cruz Biotechnology; 1∶500), mouse-anti-Rac1 (Upstate Biotechnology; 1∶500), rabbit-anti-Cdc42 (sc-87, Santa Cruz Biotechnology; 1∶200), mouse-anti-GAPDH (Glyceraldehyde-3-phosphate dehydrogenase; MAB374, Chemicon; 1∶20,000), peroxidase-conjugated goat-anti-mouse (Jackson Immunoresearch; 1∶5,000), and peroxidase-conjugated goat-anti-rabbit (Jackson Immunoresearch; 1∶5,000).

### Statistics

p values were calculated using the two-tailed student's t test. P<0.05 was considered significant.

## Supporting Information

Figure S1 *S. typhimurium* invasion and adhesion to mammalian cells is independent of Abl tyrosine kinases. A. HeLa cells were infected with *S. typhimurium* for 1h in the presence of the Abl inhibitor Gleevec (0–50 µM), and bacterial invasion and adhesion was measured at 1 hpi. The results are normalized with respect to untreated cells. B. Abl/Arg k.o. cells and 3T3 wildtype cells were infected with *S. typhimurium* for 1h and bacterial invasion was measured.
(0.16 MB TIF)

Figure S2 *S. typhimurium* invasion into HeLa cells is independent of CrkI/II. A. HeLa cells were treated with Crk or control (c) siRNA. Cell lysates immunoblotted with an anti-CrkI/II-antibody showed decreased protein levels compared to control siRNA-treated cells. GAPDH was used as loading control. B. HeLa cells treated with CrkI/II and control siRNA were infected with *S. typhimurium* for 1h and bacterial invasion was measured.
(0.23 MB TIF)

Table S1 Host factors that are required for invasion of *P. aeruginosa* into S2 cells. RNAi-mediated depletion of the listed genes decreased *P. aeruginosa* invasion into S2 cells by at least 33% compared to invasion into untreated cells. Gene accession numbers are from Flybase (http://flybase.bio.indiana.edu).
(0.06 MB DOC)

Table S2 RNAi-mediated depletion of the listed host factors did not affect *P. aeruginosa* invasion into S2 cells. RNAi-mediated depletion of the listed genes did not reduce *P. aeruginosa* invasion by more than 32% compared to invasion into untreated cells. Gene accession numbers are from Flybase (http://flybase.bio.indiana.edu).
(0.08 MB DOC)
