# Supplementary figures and images for: RNAi Screen Reveals an Abl Kinase-Dependent Host Cell Pathway Involved in Pseudomonas aeruginosa Internalization
Source: PLoS Pathog. 2008 Mar 21;4(3):e1000031. doi: 10.1371/journal.ppat.1000031 (PMC2265438; doi:10.1371/journal.ppat.1000031)

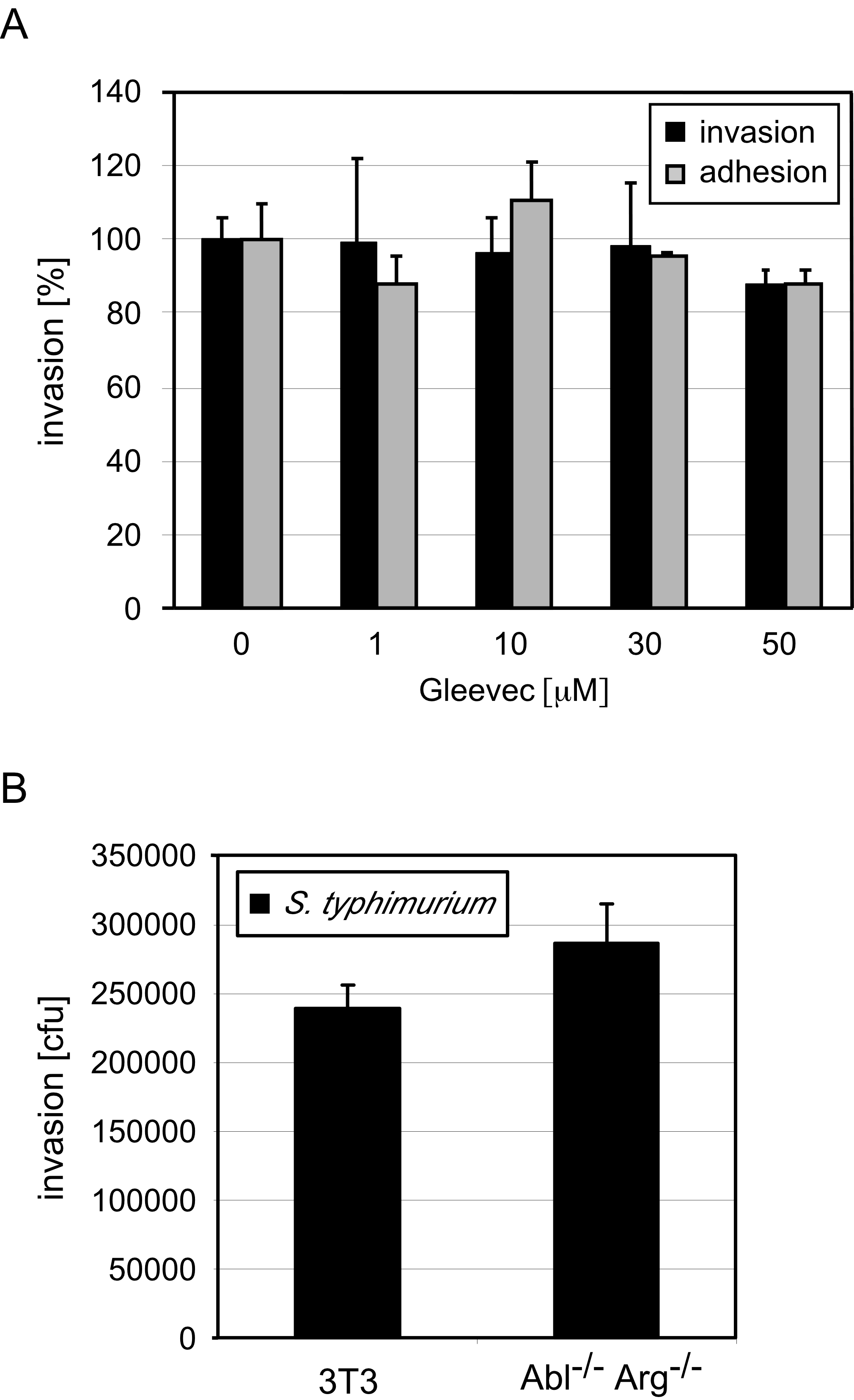

Supplement: Figure S1 — S. typhimurium invasion and adhesion to mammalian cells is independent of Abl tyrosine kinases. A. HeLa cells were infected with S. typhimurium for 1h in the presence of the Abl inhibitor Gleevec (0–50 µM), and bacterial invasion and adhesion was measured at 1 hpi. The results are normalized with respect to untreated cells. B. Abl/Arg k.o. cells and 3T3 wildtype cells were infected with S. typhimurium for 1h and bacterial invasion was measured. (0.16 MB TIF) [file ppat.1000031.s001.tif]

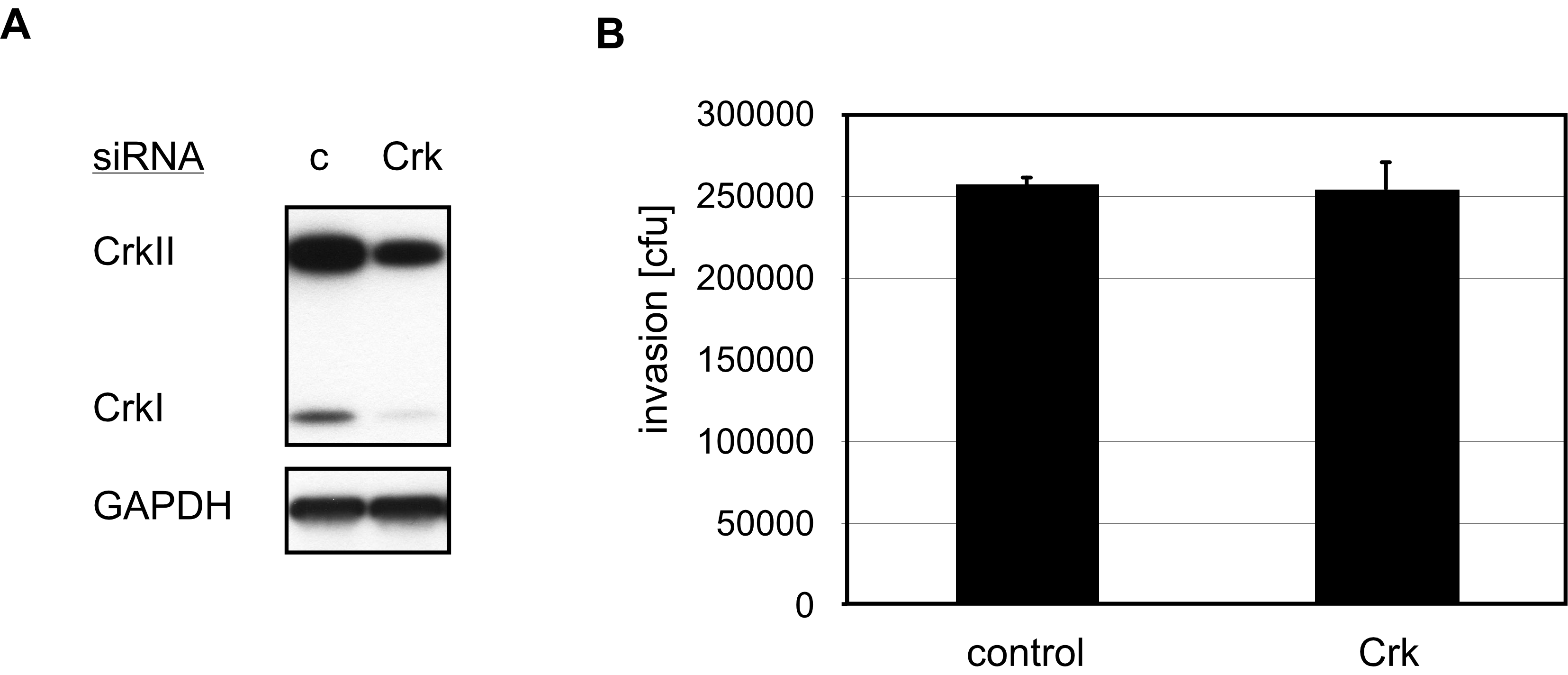

Supplement: Figure S2 — S. typhimurium invasion into HeLa cells is independent of CrkI/II. A. HeLa cells were treated with Crk or control (c) siRNA. Cell lysates immunoblotted with an anti-CrkI/II-antibody showed decreased protein levels compared to control siRNA-treated cells. GAPDH was used as loading control. B. HeLa cells treated with CrkI/II and control siRNA were infected with S. typhimurium for 1h and bacterial invasion was measured. (0.23 MB TIF) [file ppat.1000031.s002.tif]
