# Supplementary material for: RNAi Screen Reveals an Abl Kinase-Dependent Host Cell Pathway Involved in Pseudomonas aeruginosa Internalization
Source: PLoS Pathog. 2008 Mar 21;4(3):e1000031. doi: 10.1371/journal.ppat.1000031 (PMC2265438; doi:10.1371/journal.ppat.1000031)
Supplement: Table S1 — Host factors that are required for invasion of P. aeruginosa into S2 cells. RNAi-mediated depletion of the listed genes decreased P. aeruginosa invasion into S2 cells by at least 33% compared to invasion into untreated cells. Gene accession numbers are from Flybase (http://flybase.bio.indiana.edu). (0.06 MB DOC) [file ppat.1000031.s003.doc]

**Table S1: Host factors that are required for invasion of *P. aeruginosa* into S2 cells.**

| **Gene** | Accession Number |
| --- | --- |
| α-Actinin | CG4376 |
| Abi | CG9749 |
| Abl | CG4032 |
| AcGAP | CG13345 |
| ADF/cofilin-like | CG6873 |
| Aip1 | CG10724 |
| Akt | CG4006 |
| Anillin | CG2092 |
| Capping protein beta | CG17158 |
| Crk | CG1587 |
| Cyclin-dependent kinase 5 | CG8203 |
| Dah | CG6157 |
| Disabled | CG9695 |
| Drebrin-like | CG10083 |
| Drk | CG6033 |
| Kette | CG5837 |
| Lethal (2) Giant Larvae | CG2671 |
| Nullo | CG14426 |
| Arp2/3 complex (p20 subunit) | CG5972 |
| Pak | CG10295 |
| PI3K 21B | CG2699 |
| PI3K 92E | CG4141 |
| Pkn | CG2049 |
| Rab5 | CG3664 |
| Rho BTB | CG5701 |
| RhoL | CG9366 |
| Scar | CG4636 |
| WASP | CG1520 |
