# Supplementary material for: RNAi Screen Reveals an Abl Kinase-Dependent Host Cell Pathway Involved in Pseudomonas aeruginosa Internalization
Source: PLoS Pathog. 2008 Mar 21;4(3):e1000031. doi: 10.1371/journal.ppat.1000031 (PMC2265438; doi:10.1371/journal.ppat.1000031)
Supplement: Table S2 — RNAi-mediated depletion of the listed host factors did not affect P. aeruginosa invasion into S2 cells. RNAi-mediated depletion of the listed genes did not reduce P. aeruginosa invasion by more than 32% compared to invasion into untreated cells. Gene accession numbers are from Flybase (http://flybase.bio.indiana.edu). (0.08 MB DOC) [file ppat.1000031.s004.doc]

**Table S2: RNAi-mediated depletion of the listed genes did not affect *P. aeruginosa* invasion into S2 cells.**

| **Gene** | Accession Number |
| --- | --- |
| Cappucino | CG3399 |
| Capulet | CG5061 |
| Cdc42 | CG12530 |
| Ciboulot | CG4944 |
| Cofilin | CG4254 |
| Coracle | CG11949 |
| Cortactin | CG3637 |
| D-carmil | CG1399 |
| Diaphanous | CG1768 |
| Dlar | CG10443 |
| Drok | CG9774 |
| Enabled | CG15112 |
| Fascin | CG1536 |
| Filamin | CG3937 |
| Fimbrin | CG8649 |
| Forked | CG5424 |
| Formin | CG14622 |
| Gelsolin | CG1106 |
| Genghis Kahn | CG4012 |
| Hemipterous | CG4353 |
| HSPC300 | CG30173 |
| Kelch | CG7210 |
| Lim kinase | CG1848 |
| Merlin | CG14228 |
| Moesin | CG10701 |
| Mtl | CG5588 |
| Mushroom bodies tiny | CG18582 |
| Myoblast city | CG10379 |
| Nck | CG3727 |
| Ovarian tumor | CG12743 |
| PI3K 59F | CG5373 |
| PI3K 68D | CG11621 |
| Pod-1 | CG4532 |
| Pp2A | CG13383 |
| Profilin | CG9553 |
| Quail | CG6433 |
| Rac1/2 | CG2248 |
| Rho1 | CG8416 |
| Rhophilin | CG8497 |
| Sanpodo | CG1539 |
| Slingshot | CG6238 |
| Spire | CG10076 |
| Sra-1 | CG4931 |
| Talin | CG6831 |
| Trio | CG18214 |
| Twinfillin | CG3172 |
| Vav | CG7893 |
| Villin-like | CG1141 |
| Vinculin | CG3299 |
